# Controlling the oxidation state of molybdenum oxide nanoparticles prepared by ionic liquid/metal sputtering to enhance plasmon-induced charge separation†

**DOI:** 10.1039/d0ra05165a

**Published:** 2020-08-03

**Authors:** Kazutaka Akiyoshi, Tatsuya Kameyama, Takahisa Yamamoto, Susumu Kuwabata, Tetsu Tatsuma, Tsukasa Torimoto

**Affiliations:** Graduate School of Engineering, Nagoya University Furo-cho, Chikusa-ku Nagoya 464-8603 Japan torimoto@apchem.nagoya-u.ac.jp; Graduate School of Engineering, Osaka University 2-1 Yamada-oka Suita Osaka 565-0871 Japan; Institute of Industrial Science, The University of Tokyo 4-6-1 Komaba, Meguro-ku Tokyo 153-8505 Japan

## Abstract

Nanoparticles composed of molybdenum oxide, MoO_*x*_, were successfully prepared by room-temperature ionic liquid (RTIL)/metal sputtering followed by heat treatment. Hydroxyl groups in RTIL molecules retarded the coalescence between MoO_*x*_ NPs during heat treatment at 473 K in air, while the oxidation state of Mo species in MoO_*x*_ nanoparticles (NPs) could be modified by changing the heat treatment time. An LSPR peak was observed at 840 nm in the near-IR region for MoO_*x*_ NPs of 55 nm or larger in size that were annealed in a hydroxyl-functionalized RTIL. Photoexcitation of the LSPR peak of MoO_*x*_ NPs induced electron transfer from NPs to ITO electrodes.

## Abstract 2

MoO_*x*_ NPs, prepared by sputtering Mo metal on a room-temperature ionic liquid (RTIL) followed by heating in air, produced anodic photocurrents with the excitation of their LSPR peak.

## Graphical abstract

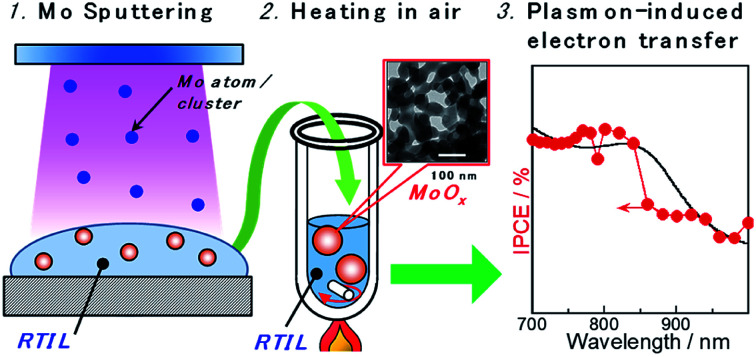

## Introduction

Light irradiation of plasmonic nanoparticles (NPs) can induce a collective oscillation of free carriers, so-called localized surface plasmon resonance (LSPR), that can lead to the generation of strong electromagnetic fields at the surfaces.^1^ When plasmonic NPs were combined with a semiconductor such as TiO_2_ or ZnO, plasmon-induced charge separation (PICS),^2–5^ by which electrons or holes moved from the NPs to the semiconductor, was observed. Recently, there have been some reports published on PICS from plasmonic NPs to metallic conductors such as graphene substrates^6–9^ and indium tin oxide (ITO) electrodes.^10–15^ This phenomenon has been intensively investigated for the development of photovoltaics, photocatalysts, and biosensors.^2–5,16–18^ So far, most research on PICS has been carried out with metal NPs showing intense LSPR peaks, such as Au and Ag.

Recently, much attention has been given to the development of novel plasmonic nanostructures with less expensive materials. Among the various materials for possible application, metal oxides have been promising because the position of LSPR peaks was reported to be easily controlled over a wide wavelength region from visible light to near-IR light by changing the composition.^19–21^ For example, several kinds of semiconductor/metal oxide systems including TiO_2_/MoO_3_,^22^ TiO_2_/ITO,^23^ and SnO_2_/ITO^24^ have been reported to exhibit PICS. However, the preparation methods have not yet been completely optimized. Furthermore, the efficiency of PICS has been low at a longer wavelength because the energy of absorbed photons seemed to be too low for photogenerated electrons to overcome the Schottky barrier height at the metal–semiconductor interface.^4,25–29^

On the other hand, room-temperature ionic liquids (RTILs) have been fascinating media to prepare nanostructured materials because the obtained structures were quite stable without the addition of any stabilizing agents.^30–39^ We have reported strategies to prepare metal and alloy NPs by sputtering a metal onto RTILs under a reduced pressure, the RTIL/metal sputtering technique, with the use of unique features of RTILs such as extremely low vapor pressure and high thermal stability.^40–42^ This technique enabled clean preparation of plasmonic NPs such as Au,^43^ Ag,^44^ Cu,^45^ AgAu,^46^ and AuCu.^47^ Furthermore, when transition metals with relatively negative redox potentials were sputter-deposited on RTILs, corresponding metal oxides, such as indium oxide,^48^ molybdenum oxide, and tungsten oxide,^49^ were formed *via* oxidation with O_2_ or H_2_O contained in the RTIL as an impurity. However, the optical properties of thus-obtained NPs have not been reported.

In this study, we controlled the oxidation state of molybdenum oxide NPs prepared by the RTIL/metal sputtering technique and we investigated their LSPR properties. Furthermore, their PICS behaviour was clarified by irradiating thus-obtained NPs immobilized on ITO electrodes.

## Experimental

### Preparation of molybdenum oxide NPs

RTILs of 1-hydroxyethyl-3-methylimidazolium tetrafluoroborate (HyEMI-BF_4_) and 1-ethyl-3-methylimidazolium tetrafluoroborate (EMI-BF_4_) were dried at 373 K for 3 h with vigorous stirring under a vacuum condition before use. The contents of water were determined to be 560 ppm and 90 ppm for HyEMI-BF_4_ and EMI-BF_4_, respectively, by the Karl Fischer titration method (Kyoto Electronics Manufacturing, MKC-610). An RTIL (0.60 cm^3^) was spread on a glass plate (10 cm^2^) that was horizontally set in the sputter coater (Sanyu Electron Co. Ltd., SC-701HMCII). The surface of the RTIL was located at a distance of 25 mm from the Mo target (99.99% in purity). Sputter deposition of Mo on RTILs was carried out for 1 h with a discharge current of 10, 20, 30, or 40 mA under argon pressure of 3.0 Pa. The as-sputter-deposited NPs were further oxidized to produce MoO_*x*_ NPs by annealing RTILs containing NPs at 473 K for various times, typically 30 min, in air with relative humidity of 40–50%.

Extinction spectra of NPs in RTILs were measured by a spectrophotometer (Agilent Technologies, Agilent 8453) in which a quartz cell with a light path length of 0.10 mm was used, and a blank solution for the measurements was an RTIL without metal sputtering. The concentration of Mo deposited in an RTIL was determined by X-ray fluorescence spectroscopy (Rigaku, EDXL300). Structural morphology of the NPs was observed by using a transmission electron microscope (TEM; HITACHI, H-7650) operated with an acceleration voltage at 100 kV. A Cs-corrected HR-STEM (JEOL, ARM-200F) with an acceleration voltage of 200 kV was used to acquire high-resolution TEM images. Samples for TEM observation were prepared by dipping a copper grid with amorphous carbon overlayers (Oken Shoji, # 10-1012) into the RTIL containing NPs. The excess amount of RTIL was rinsed with acetonitrile followed by drying. X-ray diffraction (XRD) patterns were measured with an X-ray diffractometer (Rigaku, SmartLab-3K) using Cu Kα radiation. The samples for the XRD measurements were prepared by separating the NPs from RTILs. A large amount of acetonitrile was added to NP-containing RTILs. The resulting mixture was centrifuged, and the precipitates were set onto a low-background silicon sample holder.

### Photoelectrochemical measurements

MoO_*x*_ NPs were isolated by centrifugation at 15 000 rpm for 5 min and washed with acetonitrile several times. The thus-obtained NPs were finally dispersed in acetonitrile. The MoO_*x*_ NP dispersion was spread on an ITO substrate, followed by drying. The resulting electrode with NPs of 9.4 × 10^−6^ mol cm^−2^ as Mo atoms was used as a working electrode for photoelectrochemical measurements, and an Ag/AgCl electrode and a Pt wire were used as a reference electrode and a counter electrode, respectively. The MoO_*x*_ NP-immobilized ITO electrodes, ITO/MoO_*x*_ NPs, were irradiated with a Xe lamp (*λ* > 350 nm) in a 0.5 mol dm^−3^ Na_2_SO_4_ aqueous solution (pH 6.5), the light intensity of which was 0.37 W cm^−2^. Action spectra of the photocurrent were obtained by passing the light of the Xe lamp through a monochromator (Jasco, CT-10), in which the incident photon-to-electron conversion efficiency (IPCE) was plotted as a function of the wavelength of irradiated monochromatic light.

## Results and discussion

### Preparation of molybdenum oxide NPs showing LSPR

We previously reported that sputter deposition of Mo on EMI-BF_4_ produced NPs composed of molybdenum oxide with various Mo valences.^49^ However, their optical properties were not clarified. Thus, at first, we investigated the influence of the kind of RTILs used on the optical properties of deposited NPs.

**Fig. 1:**
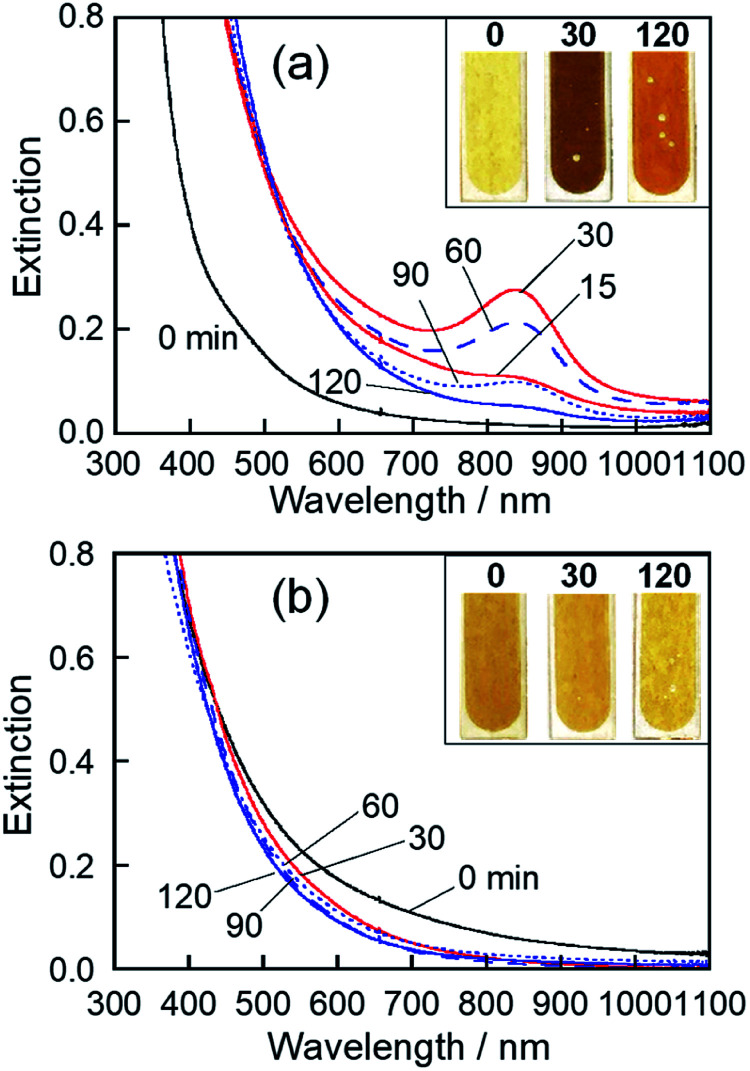
Extinction spectra of NPs in HyEMI-BF_4_ (a) and EMI-BF_4_ (b) prepared by Mo sputtering (discharge current of 30 mA) and their changes with heating at 473 K for various times in air. Photographs of the solutions with heating times of 0, 30, and 120 min are shown in the insets of the corresponding panels.

The colour of the Mo-deposited HyEMI-BF_4_ solution was remarkably changed by heat treatment from yellow brown at 0 min, dark brown at 30 min, to light brown at 120 min. As shown in Fig. 1a, the NPs in HyEMI-BF_4_ solution exhibited a structureless spectrum just after Mo sputter deposition, but the following heat treatment at 473 K resulted in the development of a new peak at *ca.* 840 nm. The peak intensity increased with the elapse of heating time to 30 min and then decreased with further heating. Since the LSPR peak of chemically synthesized oxygen-deficient MoO_3_ NPs was reported to be located in the wavelength range of 600–1000 nm,^50–53^ the observed peak at *ca.* 840 nm was assignable to the LSPR peak of molybdenum oxide NPs. Furthermore, heating of NPs in HyEMI-BF_4_ also increased the extinction at a wavelength of 700 nm or shorter.

**Fig. 2:**
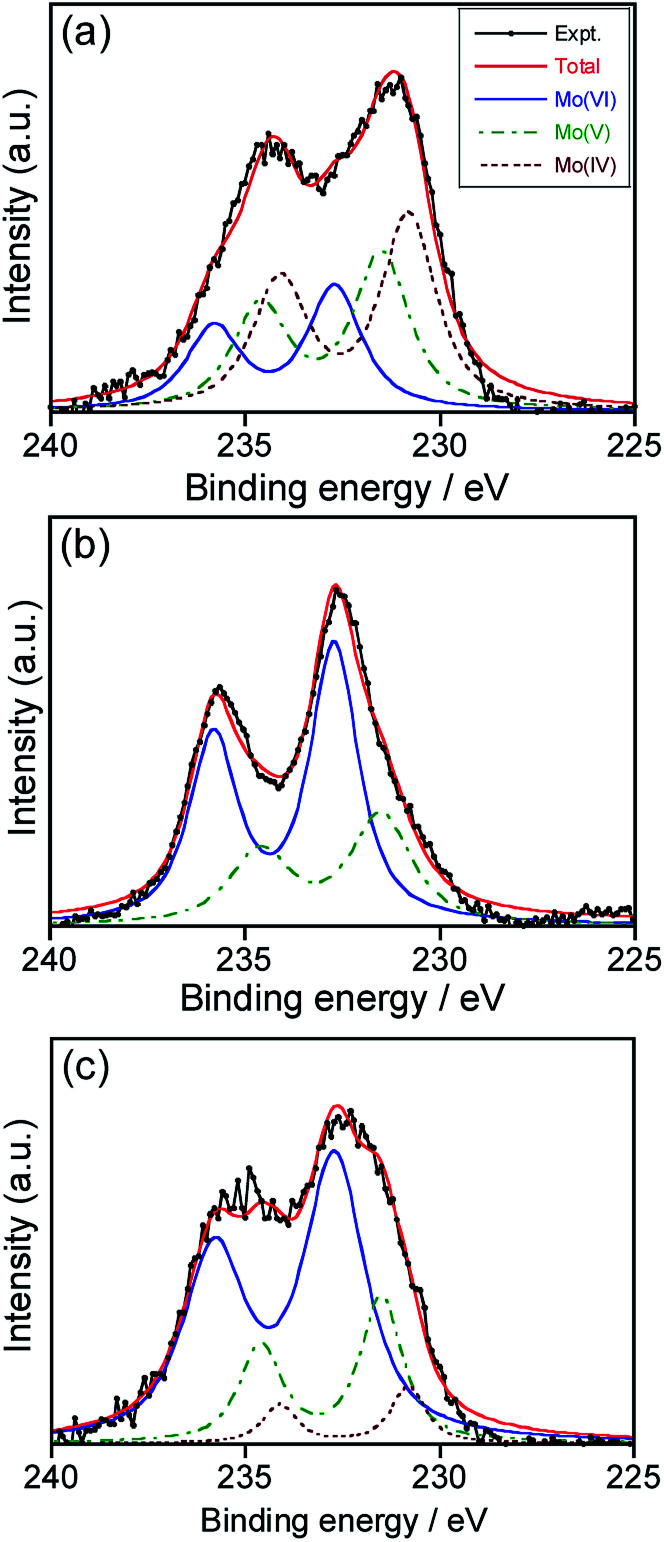
XPS spectra for Mo 3d levels of as-deposited MoO_*x*_ NPs in HyEMI-BF_4_ (a) and those after annealing at 473 K for 30 (b) and 120 min (c). The Mo sputtering was carried out with a discharge current of 30 mA.

O 1s XPS spectra (Fig. S1†) revealed the presence of Mo–O^2−^, Mo–O^−^ and Mo–OH species as well as adsorbed H_2_O on the surface (Mo–H_2_O) in deposited NPs, regardless of the heat treatment. Fig. 2 shows the Mo 3d XPS spectra of NPs deposited in HyEMI-BF_4_ with different heating times. The obtained signals were successfully deconvoluted with Mo 3d_5/2_ and 3d_3/2_ of 230.8 eV and 234.1 eV for Mo(iv), 231.5 eV and 234.6 eV for Mo(v), and 232.7 and 235.8 eV for Mo(vi), respectively.^54^ We could obtain fractions of Mo species with different oxidation states from XPS signals, as shown in Table S1.† The fraction of Mo(vi) was increased from 25.7% to 68.3% in the total Mo species with heat treatment from 0 to 120 min. No signals assignable to those at 228.3 and 231.5 eV for 3d_5/2_ and 3d_3/2_, respectively, of Mo(0)^54^ were detected. These results indicated that the metallic Mo species deposited were immediately oxidized by oxygen molecules in air and/or H_2_O molecules contained in the RTIL as an impurity and then NPs of molybdenum oxides, MoO_*x*_, were formed in HyEMI-BF_4_. Furthermore, we estimated the compositions of thus-obtained MoO_*x*_ NPs heat-treated for 0, 30 and 120 min to be *x* = 1.69, 2.29, and 3.09, respectively, from XPS signals of Mo and O.

The colour of EMI-BF_4_ sputter-deposited with Mo was slightly changed from brown to yellow-brown by heat treatment. Fig. 1b shows extinction spectra of as-deposited MoO_*x*_ NPs in EMI-BF_4_ and those heat-treated in air at 473 K for various times. A structureless spectrum was observed for as-deposited NPs in EMI-BF_4_, being similar to those in HyEMI-BF_4_. Heating Mo-deposited EMI-BF_4_ for 30 min slightly induced a blueshift of extinction spectra to the onset wavelength around 800 nm without showing an LSPR peak at around 800–900 nm, and further heat treatment up to 120 min scarcely changed the extinction spectra. XPS spectra of MoO_*x*_ NPs deposited in EMI-BF_4_ (Fig. S2†) revealed that MoO_*x*_ NPs were composed of Mo(iv), Mo(v), and Mo(vi) species, in which the fraction of Mo(vi) was roughly constant at *ca.* 20–27% regardless of the heating (Table S1†), being different from the case of the NPs in HyEMI-BF_4_. The compositions of MoO_*x*_ NPs deposited in EMI-BF_4_ were also determined from XPS signals to be *x* = 1.17, 1.61 and 2.60 for samples heat-treated for 0, 30 and 120 min, respectively. It should be noted that each *x* value for MoO_*x*_ NPs in EMI-BF_4_ was smaller than the aforementioned value of corresponding NPs in HyEMI-BF_4_, indicating the NPs in EMI-BF_4_ are less susceptible to oxidation.

As already reported in the literature, NPs consisting of metal oxide semiconductors with an oxygen-deficient composition, such as WO_3−*y*_ and MoO_3−*y*_, can absorb light through three different electronic excitation modes: (i) interband transition, that is, transition from the valence band (VB) to the conduction band (CB), (ii) transition from the VB to oxygen-deficient states formed by metal species of different valences, such as Mo(v), and (iii) polaron-induced LSPR.^55–58^ In such metal oxide semiconductors, it was reported that the optical response was extended to a longer wavelength range than that expected from the intrinsic energy gap,^59^ because oxygen vacancies formed defect states between the CB and the Fermi level (*E*_F_), narrowing their optical band gap. Considering the MoO_3_ energy gap of approximately 3 eV, corresponding to 413 nm,^55,60^ the extinction in the wavelength region of 700 nm or shorter in Fig. 1 seemed to be due to transition from the VB to oxygen vacancy states formed by Mo(iv) and Mo(v) species.

The appearance of the LSPR peak of MoO_*x*_ NPs in HyEMI-BF_4_ can be understood by considering two factors, the amount of oxygen vacancies and particle size. As mentioned above for the results shown in Fig. 2 and Table S1,† heat treatment of MoO_*x*_ NPs deposited in HyEMI-BF_4_ remarkably increased the fraction of Mo(vi) in the total Mo species with the elapse of heating time. When NPs in HyEMI-BF_4_ were heated for 30 min at 473 K, the Mo(iv) species disappeared. For such NPs, the chemical formula was calculated to be MoO_2.29_, the amount of oxygen vacancies of which was smaller than that of as-sputter-deposited NPs in EMI-BF_4_, MoO_1.61_. These results suggested that an appropriate amount of oxygen vacancies was formed with heat treatment for 30 min and could then produce free electrons in the MoO_3_ structure for the LSPR peak shown in Fig. 1a to emerge. However, the LSPR peak almost disappeared with further heat treatment, and the chemical formula of NPs changed from MoO_2.29_ to MoO_3.09_ with prolonged heating from 30 to 120 min (Table S1†). The decrease in the amount of oxygen vacancies resulted in a reduction in the number of free electrons.^61,62^ Another reason for the decrease in LSPR peak intensity was the change in size of MoO_*x*_ NPs with heating. It is well known that the position of LSPR peaks is very sensitive to the size of plasmonic nanoparticles, regardless of metals or metal oxides, being red-shifted with an increase in particle size.^63,64^

**Fig. 3:**
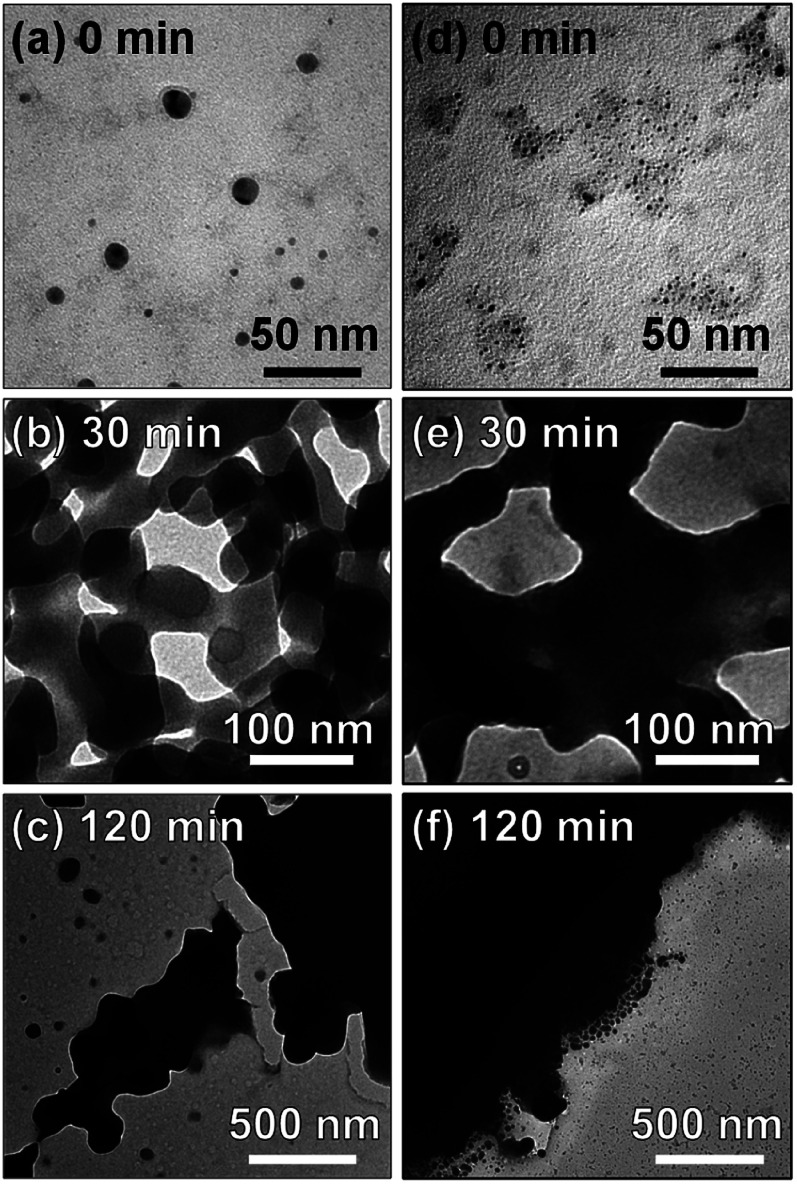
TEM images of as-deposited MoO_*x*_ NPs (a and d) and those after annealing at 473 K for 30 (b and e) and 120 min (c and f). The RTILs used were HyEMI-BF_4_ (a–c) and EMI-BF_4_ (d–f). The Mo sputtering was carried out with a discharge current of 30 mA.

Fig. 3 shows TEM images of MoO_*x*_ NPs deposited in HyEMI-BF_4_ and EMI-BF_4_. As-deposited NPs in HyEMI-BF_4_ were spherical particles with sizes of 6.8 ± 4.9 nm, which were larger than those formed in EMI-BF_4_, 2.2 ± 1.0 nm. Heating at 473 K caused significant coalescence between NPs. After heating for 30 min, the NPs in HyEMI-BF_4_ were *ca.* 65 nm in size, being much smaller than those in EMI-BF_4_, *ca.* 200 nm or more. It was reported previously that thermal oxidation of In metal in EMI-BF_4_ at 523 K produced largely aggregated NPs but that similar heat treatment in HyEMI-BF_4_ gave uniformly dispersed In_2_O_3_ NPs of 28 nm in diameter, indicating that hydroxyl groups in cationic species of RTILs were strongly adsorbed on the metal oxide surface to improve the dispersibility of NPs in the solutions.^65^ Thus, the results of the present study suggested that hydroxyl groups in HyEMI-BF_4_ molecules could be adsorbed on the MoO_*x*_ surface to retard the coalescence between NPs. The concentration of water in RTILs was also reported to significantly affect the size of In_2_O_3_ NPs formed, and the larger NPs were produced with an increase in the water concentration in RTILs.^65^ In the present study, since the water concentration in HyEMI-BF_4_, 590 ppm, was much larger than that in EMI-BF_4_, 90 ppm, a higher growth rate of MoO_*x*_ was expected in HyEMI-BF_4_, resulting in a larger number of NPs with a smaller average size. In both kinds of RTIL, the heated NPs were connected with each other to form a network structure. Furthermore, prolonged heat treatment for 120 min remarkably enlarged the size of NPs to several micrometres. Thus, it was thought that the formation of larger MoO_*x*_ NPs, >200 nm, in EMI-BF_4_ prevented the appearance of the LSPR peak.

**Fig. 4:**
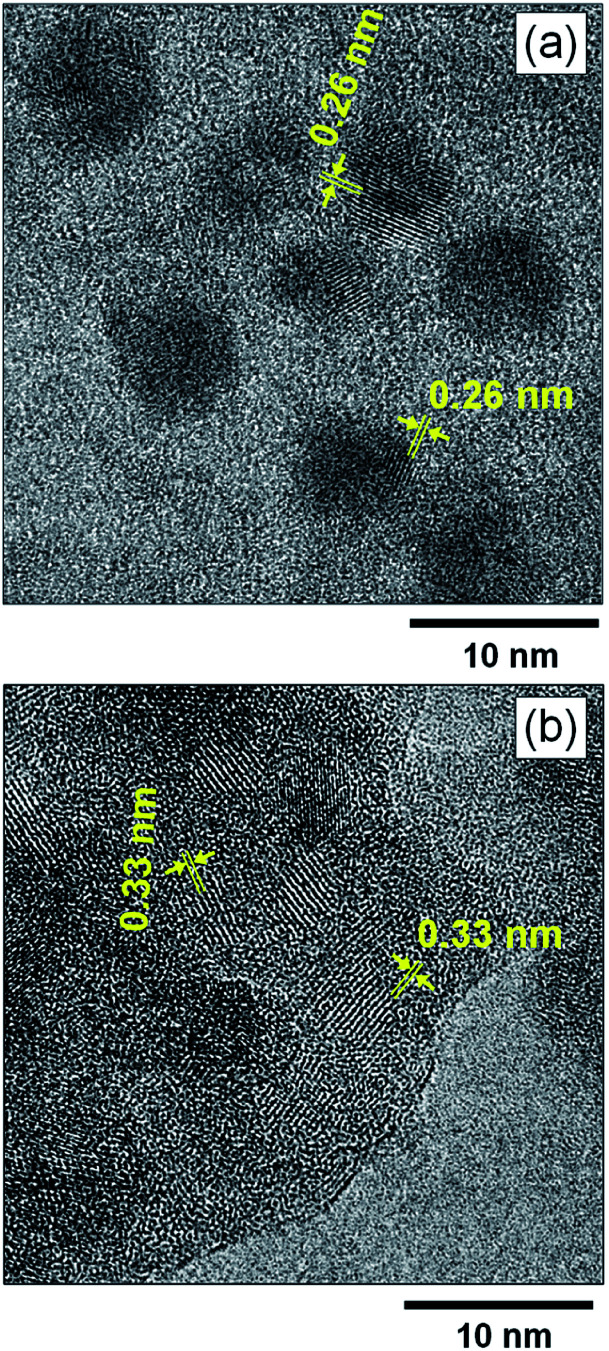
High-resolution TEM images of (a) as-deposited MoO_*x*_ NPs in HyEMI-BF_4_ and (b) those after annealing at 473 K for 30 min.

Fig. 4 shows high-resolution TEM images of MoO_*x*_ NPs formed in HyEMI-BF_4_. Lattice fringes with interplanar spacings of 0.26 and 0.33 nm were observed for as-deposited NPs in HyEMI-BF_4_ and those heat-treated at 473 K for 30 min, being assignable to (111) and (021) planes of orthorhombic MoO_3_ crystal structure, respectively. As shown in Fig. 4a, most as-deposited NPs showed lattice fringes only in a partial area of the particle. On the other hand, large particles obtained after annealing were composed of small NPs of *ca.* 1.5–6 nm in size as shown in Fig. 4b and many grain boundaries could be recognized inside the particle, indicating that each particle was polycrystalline. It should be noted that as-sputter-deposited NPs showed no characteristic diffraction peaks in the XRD patterns (Fig. S3†), though the NPs after heating at 473 K for 30 min exhibited a broad diffraction peak at *ca.* 24–27°, being assignable to orthorhombic MoO_3_ structure. These results suggested that the as-deposited NPs were composed of amorphous phase containing a very small amount of orthorhombic MoO_3_ crystal phase that could not be detected by XRD analysis but that the heat treatment increased both the fraction of orthorhombic crystal phase in NPs and their crystallinity as well as the size of coalesced NPs.

**Fig. 5:**
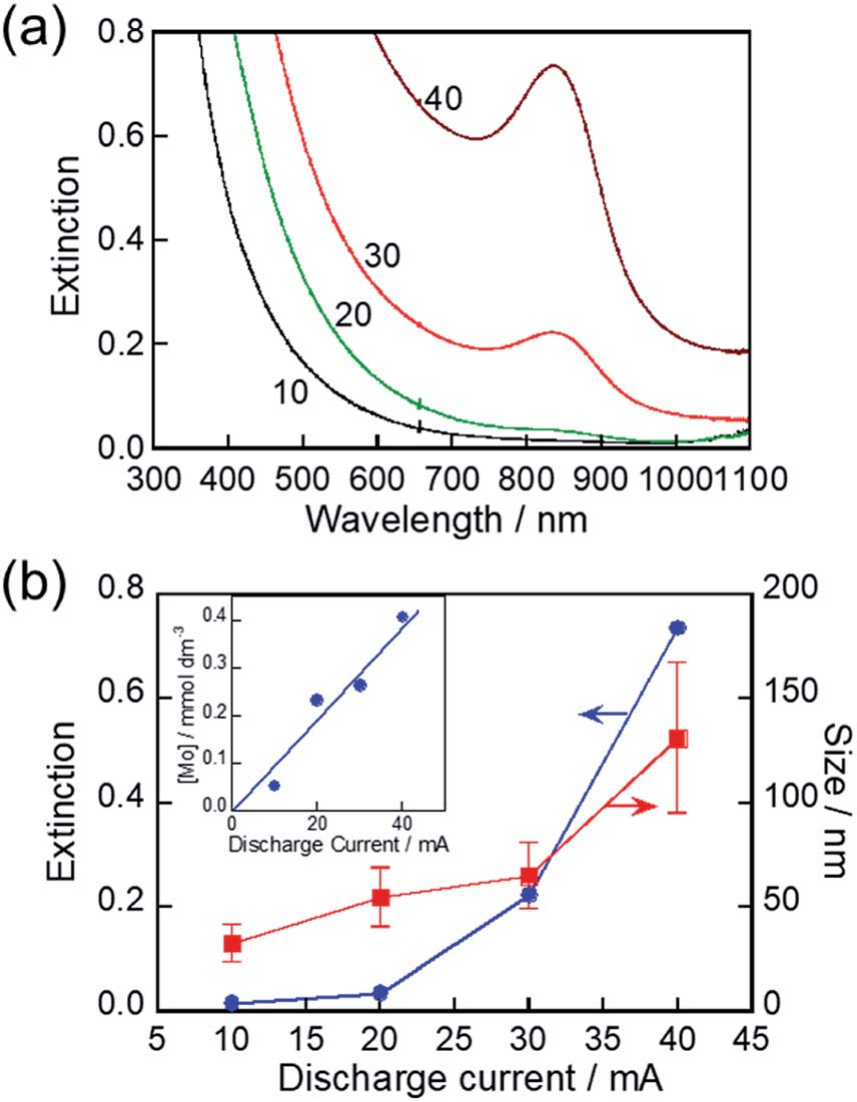
(a) Extinction spectra of NPs sputter-deposited in HyEMI-BF_4_ with various discharge currents after heating at 473 K for 30 min. Discharge currents in units of mA are shown in the panel. (b) Plots of extinction at 840 nm in panel a and size of heat-treated MoO_*x*_ NPs in the solution as a function of discharge current (inset). Relationship between concentration of Mo atoms sputter-deposited in HyEMI-BF_4_ and discharge current.

The discharge current used for the RTIL/metal sputtering technique is an important parameter for controlling the concentrations of metal species sputter-deposited in RTILs. For example, the concentrations of Au and Ag NPs deposited, as well as the size of NPs deposited, were increased with an increase in the discharge current.^66,67^ We investigated the influence of discharge current on the LSPR peak of the resulting MoO_*x*_ NPs. As-deposited NPs in HyEMI-BF_4_ exhibited a larger extinction at a wavelength shorter than 800 nm in individual spectra but did not show any LSPR peaks (Fig. S4†). The extinction at a constant wavelength was enlarged with an increase in the discharge current. The size of as-sputter-deposited MoO_*x*_ NPs increased from 2.0 nm to 17 nm with an increase in the discharge current from 10 mA to 40 mA (not shown). The concentration of Mo atoms deposited in HyEMI-BF_4_ linearly increased with increase in the discharge current (inset of Fig. 5b). On the other hand, the LSPR peak appeared at *ca.* 840 nm as shown in Fig. 5a when the NPs sputter-deposited with a discharge current of 20 mA or larger were heat-treated at 473 K for 30 min in air. Heating as-deposited NPs enlarged the particle size, accompanied by interconnection between the resulting NPs to form a network structure (Fig. S5†). Fig. 5b shows the size of heat-treated MoO_*x*_ NPs as a function of discharge current. With an increase in the discharge current from 10 mA to 40 mA, the average size of resulting NPs increased from 32 nm to 131 nm, indicating that the degree of coalescence increased with an increase in the Mo concentration in the solution. As clearly shown in Fig. 5b, an LSPR peak appeared at 840 nm for MoO_*x*_ NPs with sizes of 55 nm or larger. It was reported that spherical Au NPs smaller than *ca.* 2 nm did not show a clear LSPR peak.^68–70^ Thus, the results suggest that the minimum size for showing an LSPR peak is *ca.* 55 nm for MoO_*x*_ NPs prepared in the present study.

### Plasmon-induced charge transfer from MoO_*x*_ NPs

**Fig. 6:**
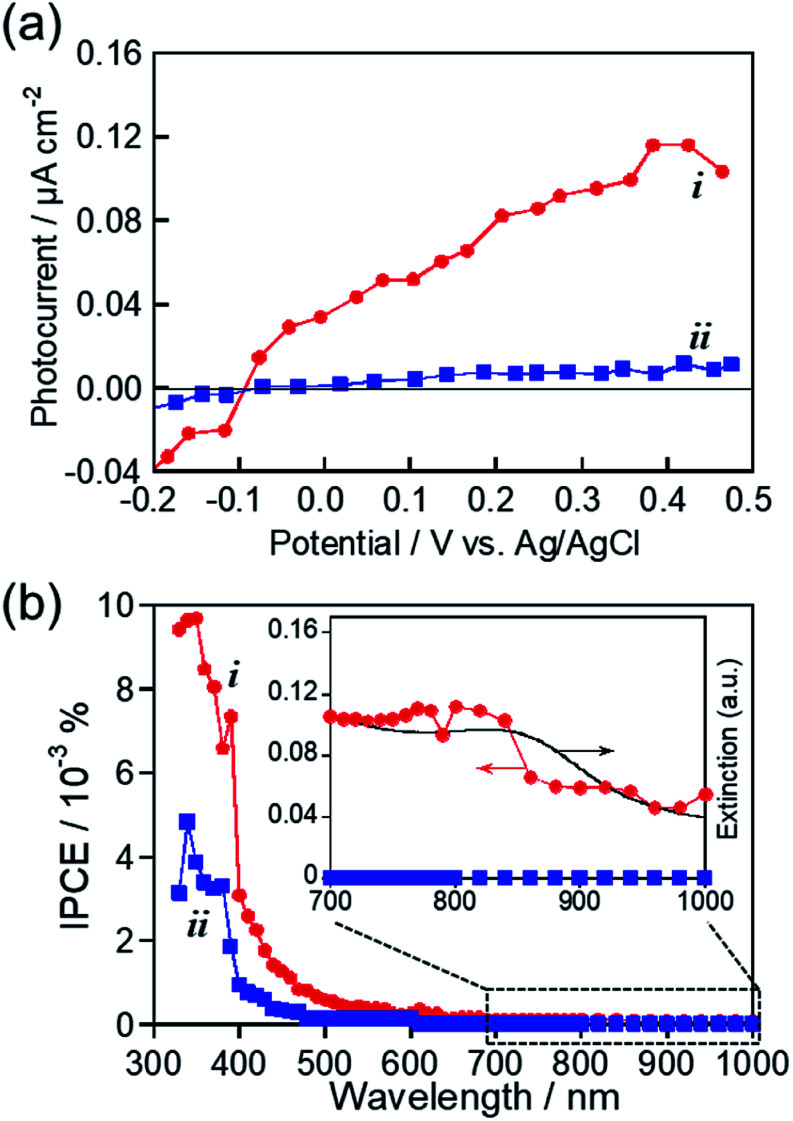
(a) Photocurrent–potential curves for ITO/MoO_*x*_ NP electrodes. The MoO_*x*_ NPs were prepared by Mo sputter deposition in HyEMI-BF_4_ (i) and EMI-BF_4_ (ii) with a discharge current of 30 mA followed by heat treatment at 473 K for 30 min in air. (b) Action spectra of anodic photocurrents in panel a under potential application at 0.5 V *vs.* Ag/AgCl.

As mentioned above, we successfully prepared MoO_*x*_ NPs with or without showing an LSPR peak in the near-IR region by changing the kind of RTIL used for sputter deposition. These NPs seem to be suitable for investigating the photoelectrochemical response of MoO_*x*_ NPs with LSPR excitation in the near-IR wavelength region. Fig. 6a shows photocurrent–potential curves of ITO/MoO_*x*_ NPs, where MoO_*x*_ NPs were prepared by Mo sputter deposition in HyEMI-BF_4_ and EMI-BF_4_ followed by heat treatment at 473 K for 30 min in air. Anodic photocurrents were observed at a more positive potential than −0.1 V *vs.* Ag/AgCl in both cases, the magnitude being increased with a positive shift of the applied potential. These results indicated that the obtained MoO_*x*_ NPs exhibited a photoresponse similar to that of an n-type semiconductor photoelectrode, as reported previously.^55,71^ Furthermore, it should be noted that the NPs prepared in EMI-BF_4_ exhibited a much lower photoactivity than that of NPs prepared in HyEMI-BF_4_, suggesting the presence of a larger amount of carrier recombination sites or trap sites in NPs.

Photocurrent action spectra were measured under potential application at 0.5 V *vs.* Ag/AgCl. As shown in Fig. 6b, photoexcitation of the interband transition of MoO_3_ with light of wavelengths shorter than *ca.* 400 nm produced a predominant fraction of anodic photocurrent in both cases, though the NPs prepared in HyEMI-BF_4_ exhibited higher IPCE values than those of NPs prepared in EMI-BF_4_. No photocurrent in the near-IR region was observed for NPs in EMI-BF_4_. However, the peak assignable to LSPR of MoO_*x*_ NPs at around 840 nm was observed in the action spectrum for NPs prepared in HyEMI-BF_4_, and the spectrum outline agreed well with that of their extinction spectrum. This suggested that PICS was observed for ITO/MoO_*x*_ NPs: the LSPR excitation produced electrons excited in the conduction band of MoO_*x*_ NPs, which were injected into the ITO electrode.^15^ It should be noted that the IPCEs obtained with excitation of the LSPR peak of MoO_*x*_ NPs were considerably lower than those with photoexcitation of the interband transition at *ca.* 400 nm or shorter. It was reported that PICS systems with photoexcitation of near-IR light, such as ITO/TiO_2_/ITO NPs^23^ and ITO/Au NPs/TiO_2_,^29^ exhibited relatively low IPCEs of similar order of magnitude to those shown in Fig. 6b. The low IPCEs obtained in the near-IR region were probably because the energy of excited electrons, generated by LSPR photoexcitation, was too low to overcome the Schottky barrier height at the metal–semiconductor interface and/or because back-electron transfer easily occurred from conducting electrodes to plasmonic NPs. Thus, we concluded that the RTIL/metal sputtering technique provides a useful strategy for preparing plasmonic MoO_*x*_ NPs and that LSPR excitation enables plasmon-induced charge transfer from thus-obtained NPs to ITO electrodes.

## Conclusions

We successfully prepared MoO_*x*_ NPs showing an LSPR peak in the near-IR region by RTIL/metal sputtering followed by heat treatment. The degree of coalescence between NPs was dependent on the kind of RTIL used, and hydroxyl groups in RTIL molecules could be adsorbed on the MoO_*x*_ surface to stabilize NPs in the RTIL. Heat treatment of as-deposited MoO_*x*_ NPs enabled control of the oxidation state of Mo species in NPs. The LSPR peak was observed in the near-IR region for MoO_*x*_ NPs of 55 nm or larger in size that were prepared in a hydroxyl-functionalized RTIL. Photoexcitation of the LSPR peak of MoO_*x*_ NPs induced electron transfer from NPs to ITO electrodes. The photoresponsivity of MoO_*x*_ in the near-IR region will be useful for developing novel plasmonic devices such as photocatalysts and solar cells.^72^ Our RTIL/metal sputtering technique coupled with heat treatment will provide a useful strategy for controlling the oxidation states of metal oxide particles for plasmonic nanostructures.

## Conflicts of interest

There are no conflicts to declare.

## Supplementary Material

RA-010-D0RA05165A-s001
