# Supplementary material for: Controlling the oxidation state of molybdenum oxide nanoparticles prepared by ionic liquid/metal sputtering to enhance plasmon-induced charge separation
Source: RSC Adv. 2020 Aug 3;10(48):28516–22. doi: 10.1039/d0ra05165a (PMC9055849; doi:10.1039/d0ra05165a)
Supplement: RA-010-D0RA05165A-s001 [file RA-010-D0RA05165A-s001.pdf]

Electronic Supplementary Material (ESI) for RSC Advances.
This journal is © The Royal Society of Chemistry 2020

Supporting Information

Controlling the Oxidation State of Molybdenum Oxide Nanoparticles Prepared by Ionic Liquid/Metal Sputtering to Enhance Plasmon-induced Charge Separation

Kazutaka Akiyoshi,[a] Tatsuya Kameyama,[a] Takahisa Yamamoto,[a] Susumu Kuwabata,[b] Tetsu Tatsuma,[c] and Tsukasa Torimoto*[a]

[a] Graduate School of Engineering, Nagoya University, Furo-cho, Chikusa-ku, Nagoya 464-8603, Japan. E-mail: torimoto@apchem.nagoya-u.ac.jp

[b] Graduate School of Engineering, Osaka University, 2-1 Yamada-oka, Suita, Osaka 565-0871, Japan.

[c] Institute of Industrial Science, The University of Tokyo, 4-6-1 Komaba, Meguro-ku, Tokyo 153-8505, Japan.

E-mail: torimoto@chembio.nagoya-u.ac.jp

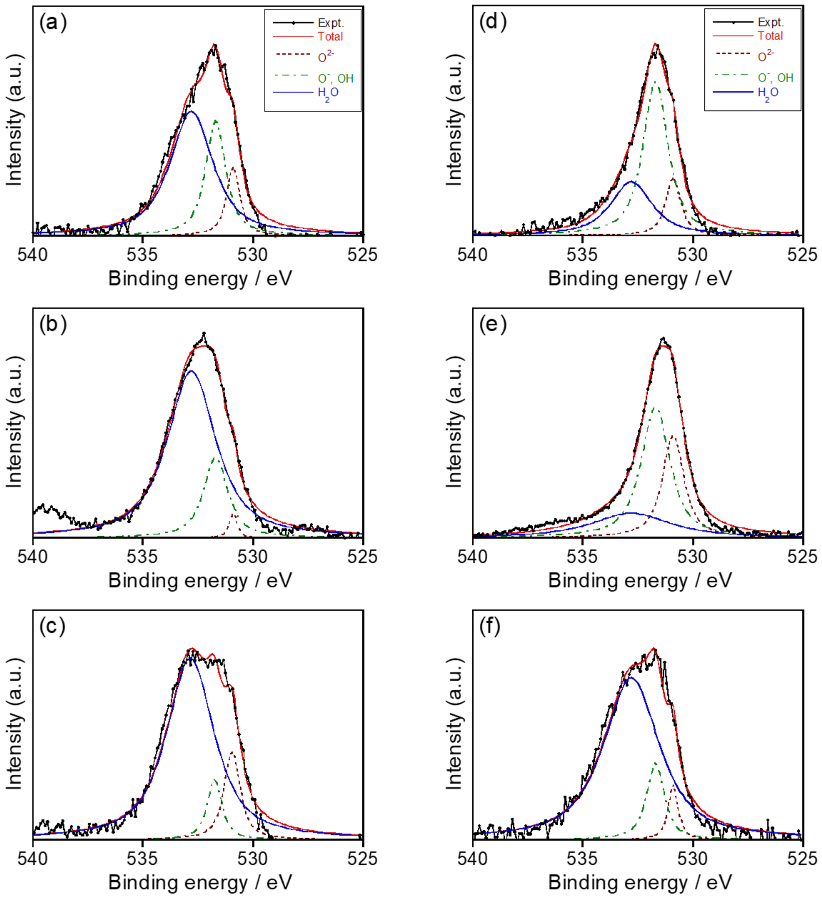

Figure S1. O 1s XPS spectra of as-deposited MoO_x NPs (a,d) and those after annealing at 473 K for 30 (b,e) and 120 min (c,f). The RTILs used were HyEMI-BF4 (a-c) and EMI-BF4 (d-f). The obtained signals were assigned with O 1s peaks of 530.9 eV for O^{2-}, 531.7 eV for O^- or OH, and 532.8 eV for H_2O.[S1] The Mo sputtering was carried out with a discharge current of 30 mA.

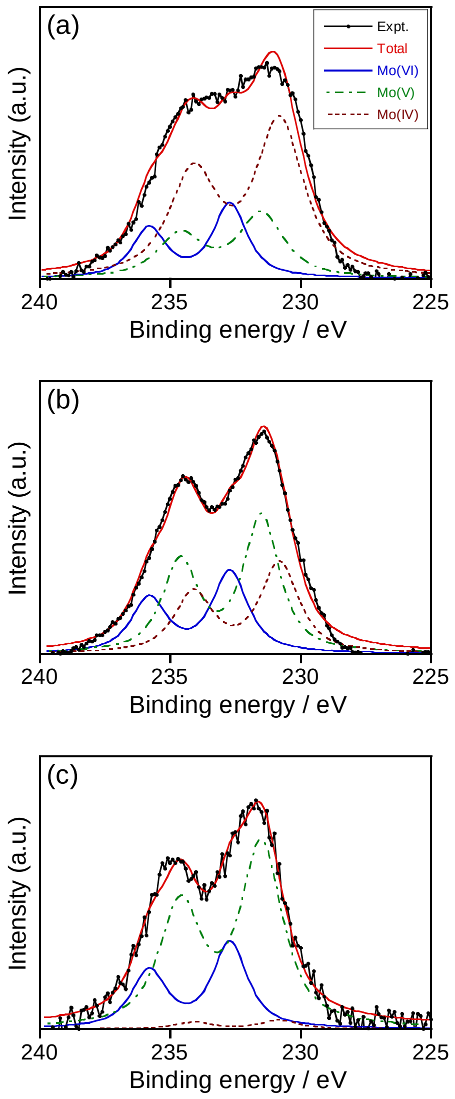

Figure S2. XPS spectra for Mo 3d levels of as-sputter-deposited MoO_x NPs in EMI-BF4 (a) and those after annealing at 473 K for 30 (b) and 120 min (c). The Mo sputtering was carried out with a discharge current of 30 mA.

Table S1. Fractions of Mo species with different oxidation states in total Mo atoms determined by peak fitting of Mo 3d XPS spectra (Figs. 2 and S2) for as-deposited MoO_x NPs in HyEMI-BF4 or EMI-BF4 and those after annealing at 473 K for 0 min, 30 min and 120 min. The chemical formulas of MoO_x NPs were estimated from XPS signals of Mo 3d and O 1s, by considering the peak areas of each band and their corresponding relative sensitivity factor.

RTIL	Heating time (min)	Mo fraction (%)			Chemical formula
		Mo(IV)	Mo(V)	Mo(VI)	
HyEMI-BF4	0	41.1	33.2	25.7	$MoO_{1.69}$
HyEMI-BF4	30	0.0	33.1	66.9	$MoO_{2.29}$
HyEMI-BF4	120	7.7	24.0	68.3	$MoO_{3.09}$
EMI-BF4	0	57.8	22.6	19.6	$MoO_{1.17}$
EMI-BF4	30	30.7	43.5	25.7	$MoO_{1.61}$
EMI-BF4	120	3.1	69.8	27.1	$MoO_{2.60}$

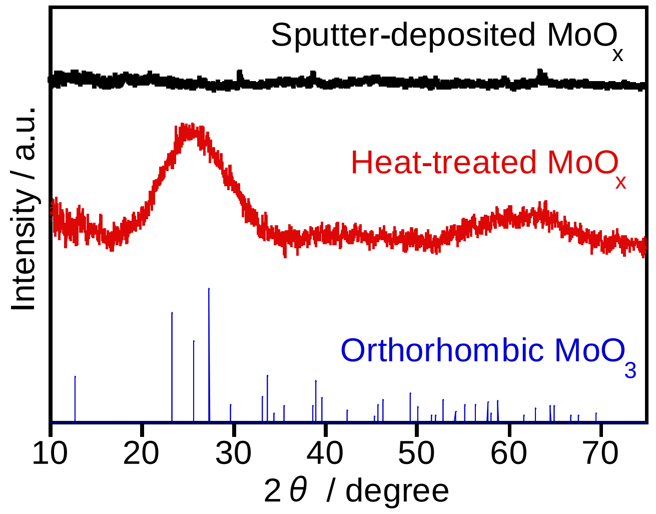

Figure S3. XRD patterns of as-deposited MoO_x NPs in HyEMI-BF4 and those after annealing at 473 K for 30 min. The standard diffraction pattern of orthorhombic MoO_3 (PDF card# 00-005-0508) is also shown. The Mo sputtering was carried out with a discharge current of 30 mA.

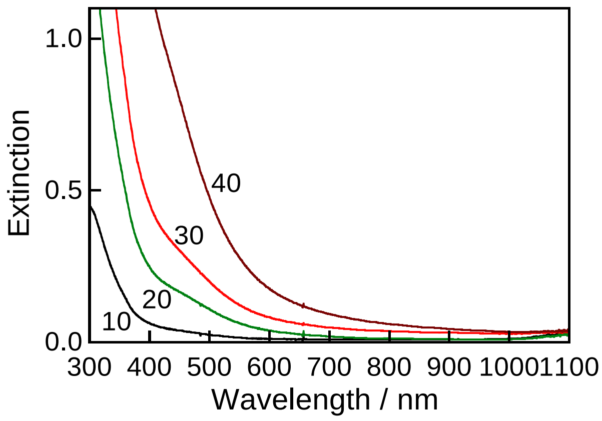

Figure S4. Extinction spectra of as-sputter-deposited NPs in HyEMI-BF4 with various discharge currents. Discharge currents in units of mA are shown in the panel.

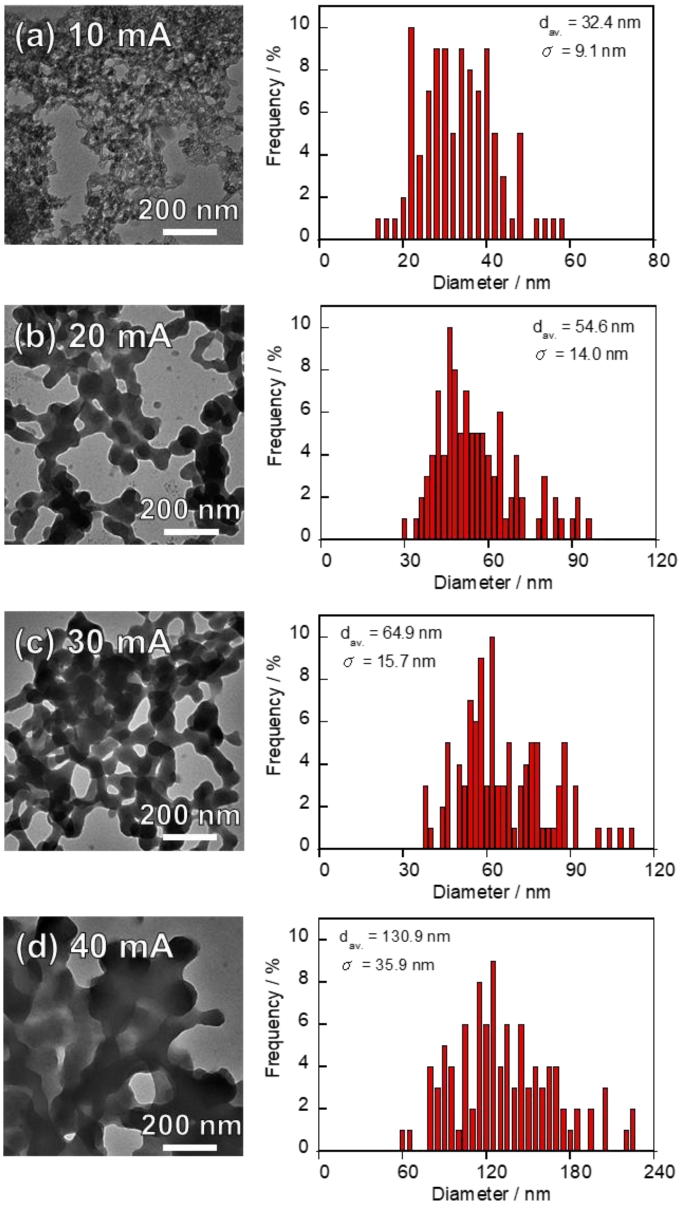

Figure S5. Representative TEM images and size distribution of MoO_x NPs deposited in HyEMI-BF4 with various discharge currents after heating at 473 K for 30 min.

Reference

(S1) A. S. Etman, H. N. Abdelhamid, Y. Yuan, L. Wang, X. Zou, and J. Sun, *ACS Omega*, 2018, **3**, 2201- 2209.
